# Long-Term Survival After Conversion Surgery and Multidisciplinary Therapy in a Case of Initially Unresectable Intrahepatic Cholangiocarcinoma

**DOI:** 10.7759/cureus.117512

**Published:** 2026-10-05

**Authors:** Shumpei Amada, Kyohei Katsuki, Yuzuru Ito, Takuji Noro, Hiroyuki Sugo

**Affiliations:** 1 Department of General Surgery, Juntendo University Nerima Hospital, Nerima, JPN

**Keywords:** conversion surgery, intrahepatic cholangiocarcinoma, multidisciplinary therapy, neoadjuvant chemotherapy, para-aortic lymph node metastases, unresectable malignancy

## Abstract

A 66-year-old woman was referred to our hospital for investigation of a 10 cm liver tumor located in the right hepatic lobe. She was diagnosed with intrahepatic cholangiocarcinoma based on a liver biopsy. Preoperative imaging strongly suggested peritoneal dissemination and para-aortic lymph node metastases. Considering the possibility of conversion surgery, the patient underwent nine cycles of systemic chemotherapy, which markedly reduced the size of the tumor. Subsequent staging laparoscopy confirmed the absence of residual peritoneal dissemination, and right hepatectomy with lymphadenectomy was performed. Fifteen months after surgery, metastatic recurrence was detected in the right lung, followed by recurrence in the left lung at 33 months. Pulmonary metastasectomy was performed for both lesions. Although adjuvant chemotherapy has been continued, the patient remains alive without evidence of recurrence at 60 months after initial presentation. Recent advances in systemic chemotherapy may provide prolonged survival for selected patients with initially unresectable and/or metastatic cholangiocarcinoma.

## Introduction

Intrahepatic cholangiocarcinoma is an aggressive malignancy with a dismal prognosis, particularly when there is lymph node metastasis or peritoneal dissemination, for which effective treatment options have traditionally been limited [1]. Nevertheless, remarkable advances in systemic chemotherapy have expanded therapeutic options for these patients and are driving a paradigm shift in the management of cancers once regarded as incurable [2,3]. In recent years, conversion surgery has emerged as a promising therapeutic strategy for selected patients with initially unresectable malignancies, particularly colorectal liver metastases and pancreatic cancer. By facilitating tumor downstaging through systemic chemotherapy, this approach has been associated with improved survival outcomes.

Here, we present a rare case of initially unresectable intrahepatic cholangiocarcinoma with suspected para-aortic lymph node metastasis and peritoneal dissemination that demonstrated an exceptional response to chemotherapy, allowing curative-intent conversion surgery. Subsequent multidisciplinary management, including two pulmonary metastasectomies for recurrent lung metastases, resulted in long-term recurrence-free survival.

## Case presentation

A 66-year-old woman presented to a local physician with upper abdominal discomfort, general fatigue, and loss of appetite. Abdominal ultrasonography revealed a tumor in the right hepatic lobe, and she was subsequently referred to our hospital for further evaluation and treatment. Her medical history was notable for hepatitis C virus (HCV) infection, which had been identified 30 years earlier but had remained untreated. The laboratory findings are summarized in Table 1. 

**Table 1:** Laboratory values

Laboratory test	Result	Reference range
White blood cell count	6500	3.6-8.9 x10^9^/L
Hemoglobin	11.4	11.1-15.2 g/dL
Platelets	347	153-346 x10^9^/L
Total bilirubin	1	0.4-1.2 mg/dL
Direct bilirubin	0.4	0.1-0.3 mg/dL
Aspartate aminotransferase (AST)	78	5-37 IU/L
Alanine aminotransferase (ALT)	18	6-43 IU/L
Alkaline phosphatase (ALP)	172	38-113 U/L
γ-glutamyl transferase (γ-GTP)	56	0-75 U/L
Lactate dehydrogenase (LD)	177	124-222 U/L
Glucose	102	65-109 mg/dL
C-reactive protein (CRP)	0.26	0.00-0.29 mg/dL
Blood urea nitrogen (BUN)	10	9-21 mg/dL
Creatinine	0.72	0.50-0.80 mg/dL
Sodium	138	135-145 mmol/L
Potassium	3.9	3.5-5.0 mmol/L
Chlorine	329	96-107 mmol/L
Prothrombin time	86	70-130 %
Activated partial thromboplastin time	29.4	23.0-36.8 seconds
Alpha-fetoprotein	25.5	0.0-10.0 ng/ml
Protein induced by vitamin K absence-II	54	-999-39.9 MAU/mL
Carcinoembryonic antigen	1.4	0.0-5.0 ng/mL
Carbohydrate antigen 19-9	19.2	0.0-37.0 U/mL
Hepatitis B surface antigen	0.001	0.000-0.005 IU/mL
Hepatitis B surface antibody	0.1	-99999-1 C.O.I.
Hepatitis B core antibody	0.1	-99999-1 C.O.I.
Hepatitis C antibody	57.6	0.0-0.9 C.O.I.
Hepatitis C virus RNA	5.1	< 1.2 LogIU/mL

The patient was positive for anti-HCV antibodies, and the HCV RNA level was 5.1 LogIU/mL. Tumor marker analysis showed mildly elevated levels of alpha-fetoprotein (AFP: 25.5 ng/mL) and protein induced by vitamin K absence-II (PIVKA-II: 54 mAU/mL), whereas carcinoembryonic antigen (CEA) and carbohydrate antigen 19-9 (CA19-9) levels were within the normal ranges. 

Contrast-enhanced computed tomography (CT) revealed a 10 cm heterogeneous, lobulated mass located in the caudal portion of the right hepatic lobe. The tumor was exposed on the liver surface and was associated with irregular thickening of the adjacent peritoneum, raising suspicion of peritoneal dissemination. In addition, enlargement of a para-aortic lymph node measuring 19 mm in diameter was observed, suggesting lymph node metastasis (Figure 1).

**Figure 1:**
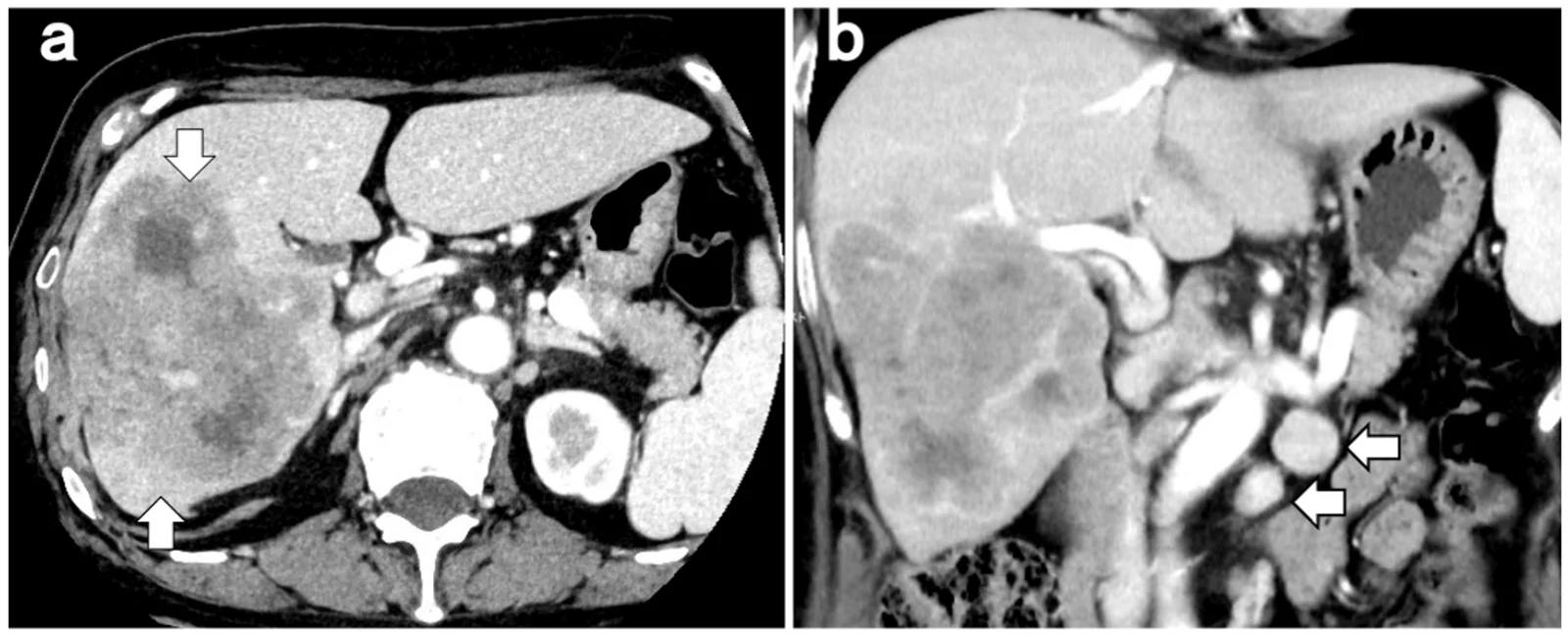
Initial CT findings CT: computed tomography CT images (a, b) revealed a 10 × 8 cm tumor in the right hepatic lobe (a, arrow) and para-aortic lymph node enlargement (b, arrows)

Magnetic resonance imaging (MRI) demonstrated findings consistent with those observed on CT, including a 10 cm lobulated tumor in the right hepatic lobe and enlarged lymph nodes in the hepatic hilum and para-aortic region. In addition, multiple irregular nodules were identified in the adjacent peritoneum on T1-weighted images. These nodules exhibited marked diffusion restriction on diffusion-weighted imaging, findings highly suggestive of peritoneal dissemination (Figure 2).

**Figure 2:**
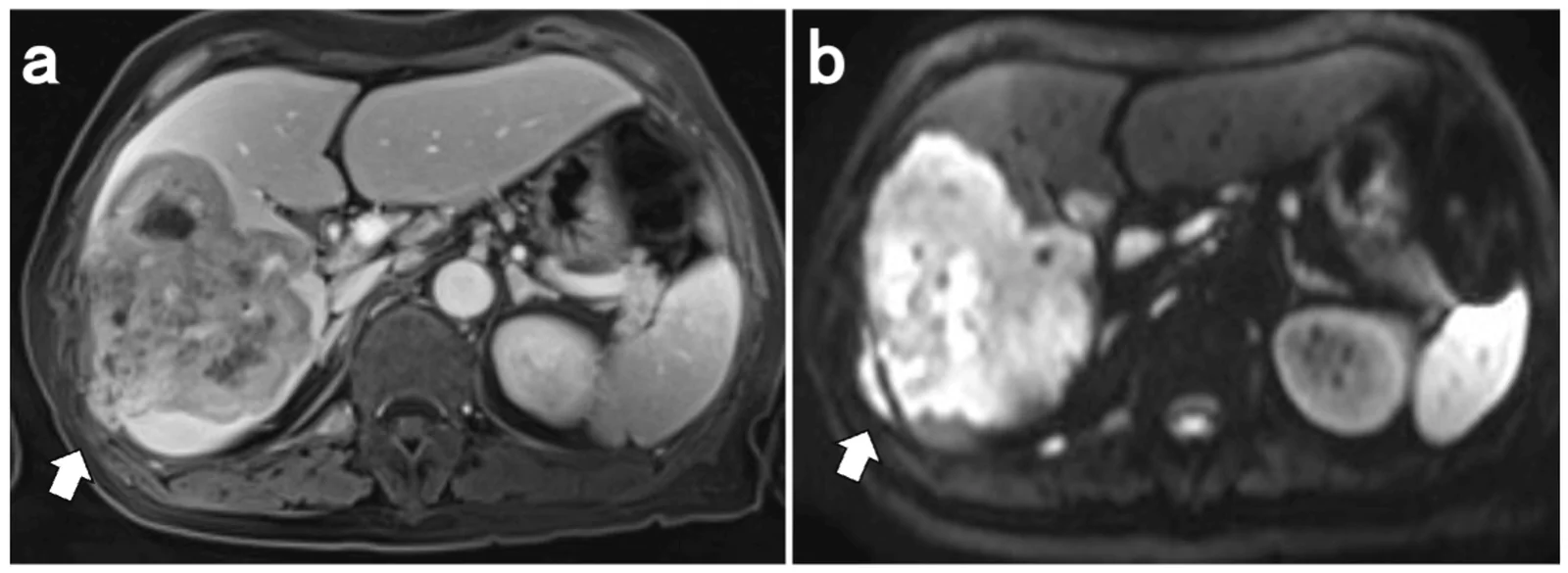
Initial MRI findings MRI: magnetic resonance imaging MRI demonstrated tumor protrusion beyond the liver surface with an indistinct border between the tumor and adjacent peritoneum on T1-weighted imaging (a, arrow). Corresponding diffusion restriction was observed on diffusion-weighted imaging (b, arrow), suggesting peritoneal dissemination

The patient was therefore diagnosed clinically with unresectable intrahepatic cholangiocarcinoma, and a liver biopsy was performed to obtain a definitive histopathological diagnosis while considering the potential for conversion surgery following systemic chemotherapy. 

Microscopic examination of the liver biopsy specimen revealed infiltrating tumor cells arranged in a trabecular pattern within a fibrotic stroma. Based on these findings, a diagnosis of intrahepatic cholangiocarcinoma was confirmed. Given the suspected para-aortic lymph node metastasis and peritoneal dissemination, the tumor was staged as cT3N1M1, clinical stage IVB [4]. Systemic chemotherapy with gemcitabine plus cisplatin (GC) was subsequently initiated. After nine cycles of chemotherapy, contrast-enhanced CT demonstrated a marked treatment response. The primary tumor decreased in size from 102 mm to 47 mm in maximum diameter, representing a reduction of more than 50%, while the suspected para-aortic lymph node decreased in size from 19 mm to 12 mm, corresponding to a reduction of more than 30% (Figure 3).

**Figure 3:**
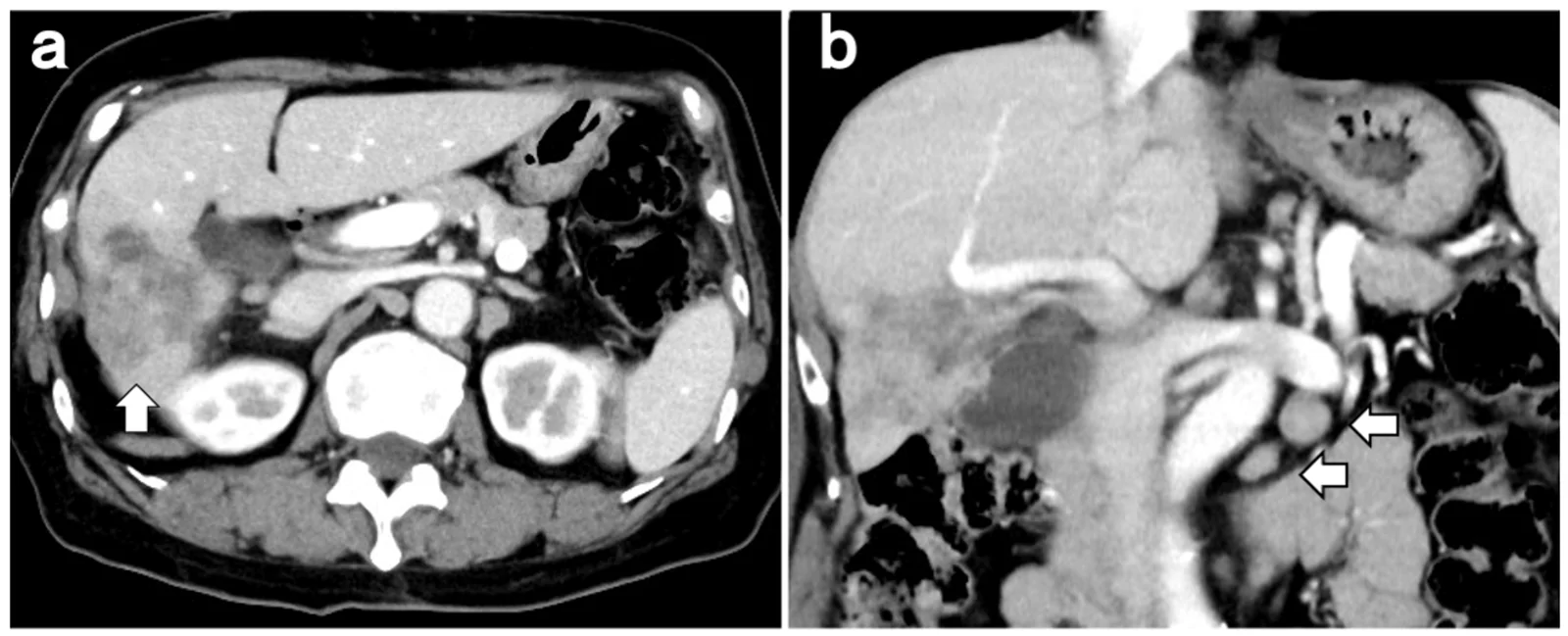
CT findings after chemotherapy CT: computed tomography Follow-up contrast-enhanced CT demonstrated a marked reduction in the size of both the primary tumor (a, arrow) and the para-aortic lymph node (b, arrows) following chemotherapy

Preoperative fluorodeoxyglucose positron emission tomography/CT demonstrated increased uptake in the primary tumor, with no abnormal uptake suggestive of extrahepatic distant metastases or peritoneal dissemination. Accordingly, the therapeutic response was assessed as a partial response according to the Response Evaluation Criteria in Solid Tumors (RECIST) criteria [5]. In addition, the previously noted peritoneal thickening, which had been considered suggestive of peritoneal dissemination, became indistinct on follow-up imaging. To evaluate the feasibility of curative resection, staging laparoscopy was subsequently performed. This revealed a whitish tumor nodule protruding through the liver surface, and several whitish nodules were also identified on the adjacent peritoneum (Figure 4).

**Figure 4:**
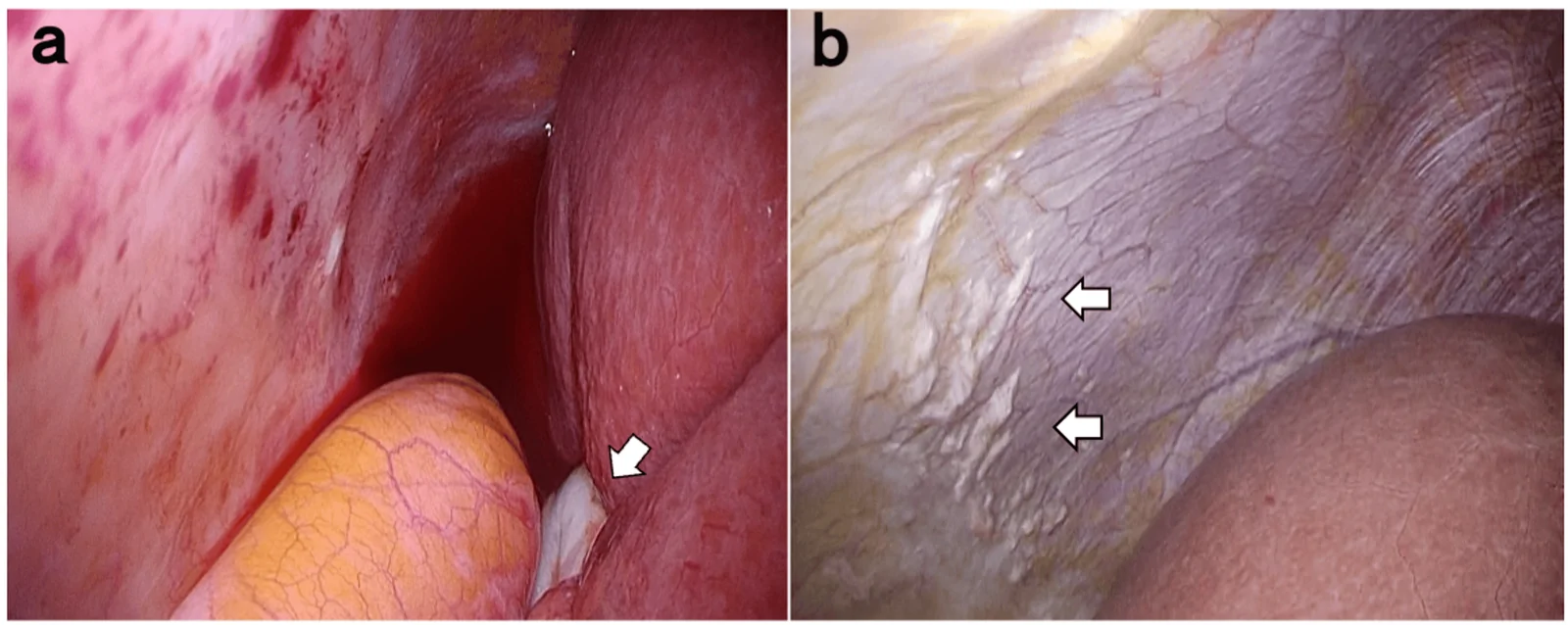
Staging laparoscopy findings A whitish lesion corresponding to the exposed hepatic tumor was observed on the liver surface (a, arrow). Multiple whitish nodules were also present on the adjacent peritoneum (b, arrow)

However, cytological examination of peritoneal lavage fluid was negative for malignancy (Class II). Furthermore, biopsy specimens obtained from the peritoneal nodules demonstrated only inflammatory and fibrotic changes, with no histopathological evidence of malignancy. Based on these findings, the patient was considered to have no remaining unresectable factors and was therefore deemed eligible for curative-intent resection. One month later, exploratory laparotomy revealed no macroscopic evidence of peritoneal dissemination. A para-aortic lymph node that had been suspected of harboring metastatic disease preoperatively was first excised and submitted for intraoperative frozen-section analysis, which revealed no evidence of malignancy. Consequently, a right hepatectomy was performed. The operation time was nine hours 35 minutes, and the intraoperative blood loss was 413 mL. The postoperative course was uneventful, and the patient was discharged on postoperative day 12. Histopathological examination of the resected specimen revealed atypical epithelial cells proliferating in a glandular pattern (Figure 5).

**Figure 5:**
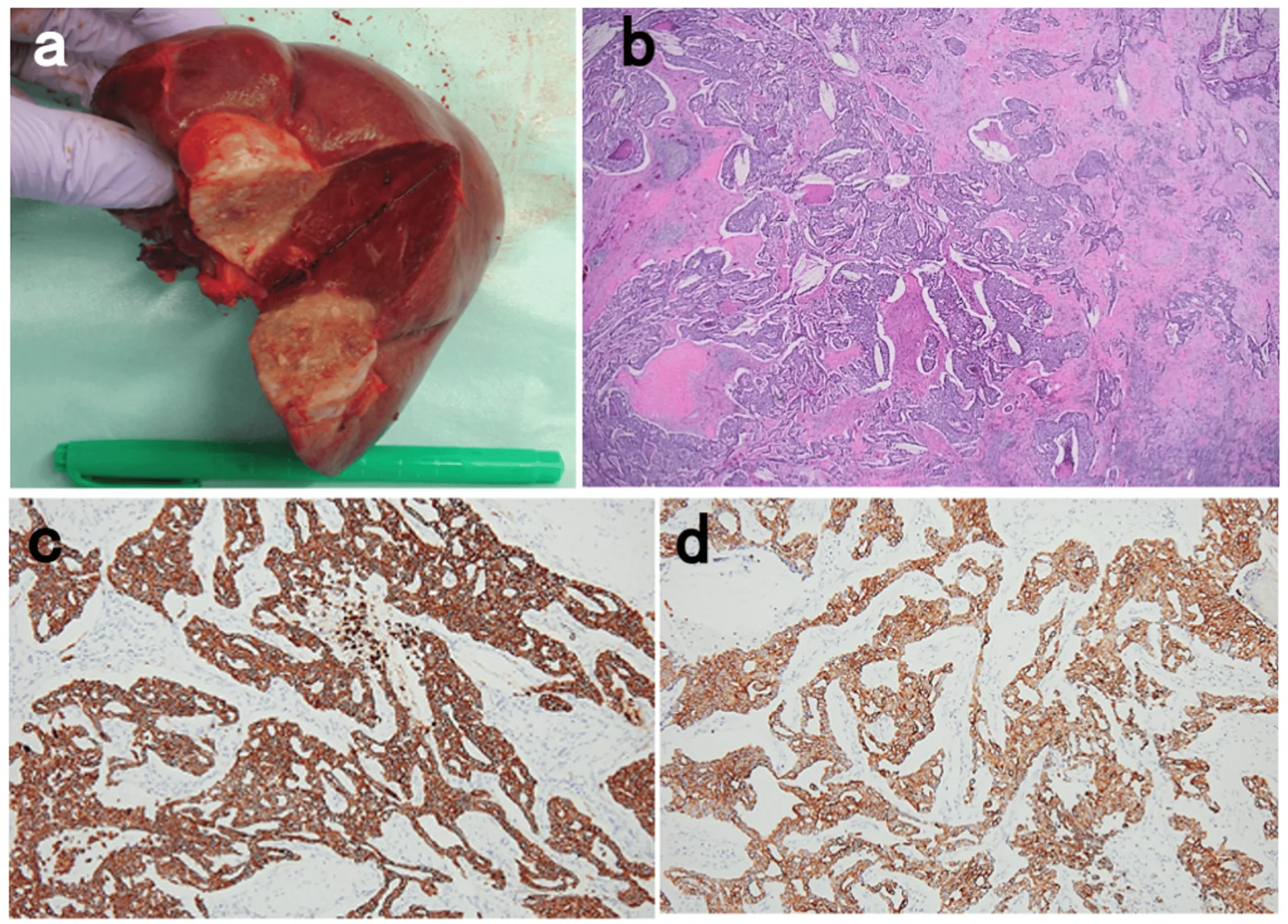
Resected specimen and histopathological findings H&E: hematoxylin and eosin; CK7: cytokeratin 7; CK19: cytokeratin 19 The macroscopic appearance of the resected specimen (a). H&E staining demonstrated atypical epithelial cells proliferating in a glandular pattern (b, ×40). Immunohistochemical staining showed diffuse positivity for CK7 (c, ×40) and CK19 (d, ×40)

Immunohistochemical staining demonstrated diffuse positivity for cytokeratin 7 (CK7) and cytokeratin 19 (CK19). Based on these findings, the tumor was diagnosed as intrahepatic cholangiocarcinoma and was pathologically staged as pT3N0M0, pStage III [3].

Postoperatively, the patient received three cycles of adjuvant GC chemotherapy. Fifteen months after the initial surgery, three metastatic nodules were detected in the right lung, and thoracoscopic partial resection of the right upper lobe was performed. Subsequently, 33 months after the initial surgery, three metastatic nodules were identified in the left lung, and thoracoscopic partial resection of the left upper lobe was undertaken (Figure 6).

**Figure 6:**
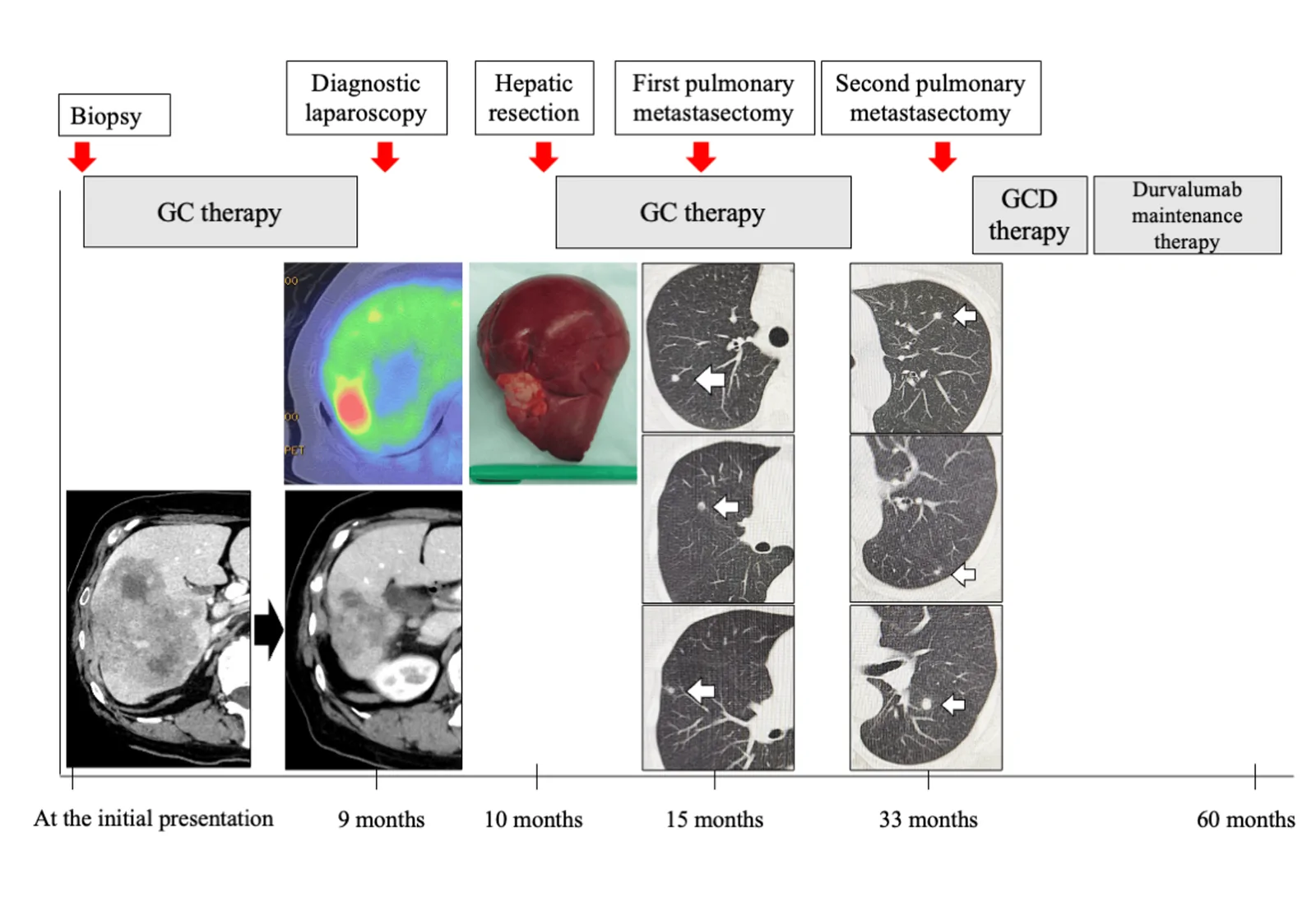
Clinical course of treatment GS: gemcitabine plus cisplatin; GCD: gemcitabine, cisplatin, and durvalumab

Histopathological examination confirmed that all of the resected pulmonary nodules were metastatic lesions originating from the intrahepatic cholangiocarcinoma. Although adjuvant chemotherapy has been continued, the patient remains alive without evidence of recurrence 60 months after initial presentation. 

## Discussion

Although the potential role of conversion surgery for intrahepatic cholangiocarcinoma has been increasingly reported in recent years, its definition and clinical significance remain controversial, and no consensus has yet been established. Furthermore, there is no universally accepted definition of unresectable biliary tract cancer, with considerable variability among countries and institutions. Particularly, the assessment of unresectability based on preoperative imaging findings, such as lymph node metastasis and vascular invasion, remains challenging. Previous studies have reported a diagnostic accuracy of only about 20% for para-aortic lymph node metastasis and a positive predictive value of 18-33% for arterial invasion [6]. These findings have led some investigators to argue that imaging findings alone should not be considered sufficient to classify a tumor as unresectable. However, it is impractical to require histopathological confirmation of all suspected unresectable factors before treatment in every patient. Moreover, unlike pancreatic cancer, for which the concept of borderline resectable disease is well established, no standardized definition of borderline resectability currently exists for intrahepatic cholangiocarcinoma. Consequently, the criteria for determining resectability in intrahepatic cholangiocarcinoma remain ill-defined, posing a challenge in selecting appropriate candidates for conversion surgery. In the present case, although the diagnosis of intrahepatic cholangiocarcinoma was confirmed by preoperative tumor biopsy, para-aortic lymph node metastasis and peritoneal dissemination were strongly suspected based on preoperative imaging findings, especially MRI. Despite the lack of histopathological confirmation of these lesions, the tumor was considered unresectable, and neoadjuvant chemotherapy was initiated with the intention of achieving conversion surgery. As a result, both the primary tumor and the suspected metastatic lymph nodes showed a favorable response to treatment. Following confirmation of the absence of peritoneal dissemination by staging laparoscopy and examination of frozen sections of the dissected lymph nodes, curative-intent hepatectomy was successfully performed. It remains uncertain whether these lesions had truly harbored metastatic cancer cells before chemotherapy or had become pathologically negative after treatment. In any event, pathological confirmation of all suspected metastatic lesions before treatment is generally impractical. Consequently, operability must be determined primarily on the basis of preoperative imaging findings, and this appears to be a reasonable approach in routine clinical practice.

The efficacy of neoadjuvant chemotherapy for borderline resectable and unresectable intrahepatic cholangiocarcinoma has been increasingly reported. Le Roy et al. reported that 39 of 74 patients with initially unresectable intrahepatic cholangiocarcinoma underwent subsequent surgical resection after preoperative chemotherapy, corresponding to a conversion rate of 53% [7]. This suggests that neoadjuvant chemotherapy may expand the indications for surgery and improve the likelihood of achieving curative resection in selected patients with advanced disease. Similarly, Kato et al. reported that 25.6% of patients with advanced biliary tract cancers, including those initially considered unresectable, became eligible for surgical resection after systemic chemotherapy [8]. Although direct comparisons among studies are difficult because the definition of unresectability has not been standardized, the prognosis of unresectable biliary tract cancer remains poor. Therefore, if neoadjuvant chemotherapy can increase the rate of curative resection, it may ultimately contribute to improved survival outcomes. 

In the present case, despite the administration of adjuvant chemotherapy after hepatectomy, pulmonary metastases developed in the right lung 15 months after the initial surgery and in the left lung after 33 months. Both lesions were treated by pulmonary metastasectomy. Surgical resection is rarely indicated for pulmonary metastases from cholangiocarcinoma because such patients generally have a poor prognosis. Previous studies have reported that pulmonary recurrence accounts for approximately 11.3% of postoperative recurrences of cholangiocarcinoma and is associated with unfavorable survival outcomes [9]. In our patient, pulmonary resection was considered appropriate because (1) no extrapulmonary recurrence was detected, (2) the metastatic burden was limited and consistent with an oligometastatic state, (3) complete resection was technically feasible, (4) the disease-free interval exceeded one year, and (5) the disease remained controllable with systemic chemotherapy. As a result, the patient remains alive without evidence of recurrence 60 months after the initial diagnosis and 38 months after the initial hepatectomy.

In the present case, the patient had a history of chronic hepatitis C but had not received antiviral therapy. Recent studies have suggested that HCV infection may be a risk factor for intrahepatic cholangiocarcinoma, potentially through its oncogenic effects, and that antiviral therapy for HCV infection may reduce the risk of recurrence [10,11]. However, antiviral therapy for the prevention of recurrence after surgery for intrahepatic cholangiocarcinoma has not yet been established, and the concomitant use of chemotherapy and antiviral therapy has also not been established. Therefore, in the present case, we prioritized chemotherapy in both the preoperative and postoperative settings and did not initiate antiviral therapy. However, more than one year has now elapsed without recurrence since the second pulmonary resection, and there may be room to reconsider the initiation of antiviral therapy as an alternative to chemotherapy in the future.　

## Conclusions

Historically, both intrahepatic cholangiocarcinoma with lymph node metastasis and pulmonary metastases from biliary tract cancer have been associated with poor outcomes. In the present case, neither lymph node metastasis nor peritoneal dissemination was ultimately confirmed pathologically. However, in clinical practice, resectability is often assessed based on preoperative imaging findings, and pathological confirmation is not always required. Therefore, some patients who are classified as unresectable based on imaging findings may potentially benefit from multidisciplinary treatment, with the possibility of improved survival outcomes. Our experience with the present case suggests that selected patients may achieve long-term disease-free survival through a multidisciplinary treatment strategy that combines systemic chemotherapy with aggressive surgical resection. Although further efforts are needed to establish standardized definitions and indications for conversion surgery, multidisciplinary treatment incorporating chemotherapy, conversion surgery, and metastasectomy may represent a therapeutic option in carefully selected patients with advanced intrahepatic cholangiocarcinoma.
